# Laparoscopic-Guided Transversus Abdominis Plane Block for Better Postoperative Pain Relief in Laparoscopic Ventral Hernia Repair: An Observational Study

**DOI:** 10.7759/cureus.94896

**Published:** 2025-10-18

**Authors:** Mohit Rakhecha, Paritosh Gupta, Ruchika Babal, Chinmay Arora

**Affiliations:** 1 Department of General Surgery and Minimal Access Surgery, Artemis Health Institute, Gurgaon, IND; 2 Department of Radiology, Artemis Health Institute, Gurgaon, IND; 3 Neurosurgery, Fortis Gurgaon, Gurgaon, IND

**Keywords:** early hospital discharge, intraperitoneal onlay mesh repair, laparoscopic guided tap block, laparoscopic ventral hernia repair, postoperative pain relief

## Abstract

Background and objective

Laparoscopic ventral hernia repair (LVHR) has been the standard procedure for abdominal wall hernia repair. However, it is associated with considerable postoperative pain attributed to the transfascial sutures and tackers used in the laparoscopic approach for this surgery. The transversus abdominis plane (TAP) block is a technique of regional anesthesia that provides analgesia to the parietal peritoneum along with the skin and muscles of the anterior abdominal wall. It helps in early recovery and reduces post-operative pain. We conducted a prospective observational single-blinded study to assess the effectiveness of this technique.

Methods

A total of 50 patients were allocated to two groups: (1) the Test Group, in which a laparoscopic TAP block was given with 0.25% bupivacaine; and (2) the Control Group, which was given the same block with normal saline. The visual analog scale (VAS) score was checked postoperatively to assess the postoperative pain score and documented at two, six, 12, 24, and 48 hours postoperatively.

Results

There were no significant differences between the Test Group (n=25) and the Control Group (n=25) in population characteristics. Postoperatively, the Test Group had lower pain scores than the Control Group (p<0.0001), and the difference was statistically significant. Test Group patients used fewer opioids than those in the Control Group after six hours postoperatively (p<0.0001). At 24 (p>0.05) and 48 hours postoperatively (p>0.05), no significant correlation in pain scores was observed.

Conclusions

We observed a significant difference in terms of immediate postoperative pain, the adequate time of the first ambulation, the need for any rescue medication, and the early readiness for discharge between patients who were given a TAP block compared to patients who did not receive it.

## Introduction

Since the introduction of laparoscopic techniques for ventral hernia repair in 1993, this approach has offered several inherent advantages. Laparoscopic techniques have certain benefits over the open technique: decreased hospital stay, faster recovery, early return to normal activities, and only a 3% recurrence rate at roughly two years postoperatively [1]. However, pain remains the most common cause of anxiety and discomfort in the immediate postoperative period, negating the benefits offered by laparoscopy [2-4]. Achieving adequate pain relief in the immediate postoperative period following laparoscopic intraperitoneal onlay mesh (IPOM) repair remains challenging, mainly due to mesh fixation involving multiple trans-fascial sutures and tackers. [5-8].

Transversus abdominis plane (TAP) blocks are a form of regional anesthesia designed to block the nerve endings located in the neurovascular plane between the internal oblique and transversus abdominis muscles [9]. Injecting a local anesthetic agent into this plane provides access to the intercostal nerves from T6 to T11 and the subcostal, ilioinguinal, and iliohypogastric nerves. These nerves traverse through the fascial plane above the transversus abdominis muscle [9-10]. In many laparoscopic procedures, TAP blocks have been employed to reduce postoperative pain and analgesic use [11-13]. In this study, we aimed to test the hypothesis that patients undergoing laparoscopic ventral hernia repair (LVHR) who receive a laparoscopic-guided TAP block with an anesthetic agent would require fewer postoperative opioids for pain management compared to those receiving a placebo.

## Materials and methods

This prospective observational study involved 50 adult patients of both sexes, aged between 18 and 79 years, who were scheduled to undergo elective laparoscopic IPOM repair under general anesthesia. The ethical approval for the study was obtained from the Artemis Health Sciences Institutional Ethics Committee (Reg No. ECR/53/Inst/HR/2013/RR-16), and informed consent was obtained from all participants before their inclusion. Patients were recruited between August 2017 and November 2019. The exclusion criteria included obstructed or strangulated ventral hernia, known history of alcoholism, opioid abuse within the last six months, insertion of abdominal drains, conversion to open surgery, psychiatric illness, unwillingness to participate, or known allergy to bupivacaine. Selected patients were allocated into two groups of 25 each based on the intervention received as per institutional protocol and not randomized: the Test Group received a laparoscopic-guided TAP block with bupivacaine, while the Control Group received a laparoscopic-guided TAP block with normal saline.

All ventral hernia repairs were performed by highly experienced laparoscopic surgeons following standard institutional surgical protocols. The same dual mesh was used in all cases and fixed with transfascial sutures and tackers. Following completion of the repair, a TAP block was administered under direct laparoscopic visualization. Patients in the Test Group received 0.25% bupivacaine at a maximum dose of 2 mg/kg, while those in the Control Group received 0.9% normal saline. Patients weighing less than 100 kg were given 50 mL in total (25 mL on each side), whereas patients weighing more than 100 kg received 60 mL in total (30 mL on each side). The block was administered bilaterally at three predetermined points along the midaxillary line with a 24-G needle under laparoscopic guidance: just below the costal margin in the subcostal region, at the triangle of Petit above the iliac crest, and at an intermediate point between the two sites. Correct infiltration was confirmed laparoscopically by visualization of Doyle’s bulge beneath the transversus abdominis muscle fibers, ensuring that the injectate was placed in the proper plane and that the peritoneum was not breached.

A standardized perioperative anesthetic protocol was followed for all patients. General anesthesia with endotracheal intubation and muscle paralysis was used. Induction was performed with intravenous propofol, and intraoperative analgesia was provided with intravenous fentanyl. Additional boluses of intravenous fentanyl 20 mcg were administered as required, and intravenous diclofenac 75 mg was given 30 minutes before the reversal of neuromuscular blockade. Immediately after surgery, all patients received intravenous paracetamol 1 g. Postoperatively, if the visual analog scale (VAS) score exceeded 4 at rest, patients were administered intravenous diclofenac 75 mg or intravenous tramadol 50 mg if diclofenac was contraindicated. The frequency of rescue analgesic administration was documented for both groups.

Postoperative assessment included VAS scores, additional analgesic requirements, time of first ambulation, willingness for discharge, and patient satisfaction scores. Pain was evaluated at two, six, 12, 24, and 48 hours by a blinded nurse in the ward using the VAS scale, ranging from 0 (no pain) to 10 (worst possible pain) [10]. Operative time was defined as the interval from the initial incision to the completion of mesh fixation or initiation of TAP block administration. Early ambulation was recorded as the time to first mobilization, while readiness for discharge was noted as the earliest time when the patient expressed willingness to go home.

All collected data were tabulated and analyzed using the SPSS Statistics software version 17.0 (IBM Corp., Armonk, NY). The normality of the data was tested before statistical analysis. Continuous variables were expressed as mean ± standard deviation (SD) or as median for skewed data, while categorical variables were presented as frequencies and percentages. Comparisons of normally distributed continuous variables between the two groups were made using the unpaired t-test, whereas the Mann-Whitney U test was used for non-normally distributed data. Categorical variables were analyzed using the chi-square test or Fisher’s exact test as appropriate. A p-value less than 0.05 was considered statistically significant.

## Results

The study involved 50 patients who were screened using the inclusion criteria. They were assigned to two study groups. Dual mesh was used in every patient, and the size of the mesh was decided according to the defect size. Two different sizes of mesh were employed: 10.8 × 15.9 cm dual mesh (a) and 15 × 21 cm dual mesh (b). The ratio of the two mesh sizes (a:b) used in both the test and control groups did not show any statistically significant difference. Each mesh was centralized over the defect and approximated to the anterior abdominal wall, followed by fixation using nonabsorbable tackers in a double crowning technique with approximately 25 to 30 tacks, irrespective of mesh size.

The Test and Control Groups were matched with respect to baseline population characteristics, and similar clinical parameters were observed between the two groups (Table 1). No statistically significant difference was noted concerning age, sex, duration of surgery, or presence of bowel adhesions. Both primary ventral and incisional hernias were included in the study, and these were distributed between the control and test groups without a significant difference (Table 2).

**Table 1:** Cohort characteristics, intraoperative parameters, postoperative time of ambulation, and time to discharge SD: standard deviation

Variables	Test Group	Control Group	P-value
Age, years, mean ± SD	51.44 ± 11.17	52.56 ± 11.73	0.731
Gender (M:F)	10:15	11:14	0.774
Duration of surgery, minutes, mean ± SD	112.20 ± 25.54	99.80 ± 20.33	0.063
Bowel adhesions present, n (%)	6 (24%)	9 (36%)	0.354
Time to first ambulate, hours, mean ± SD	10.98 ± 0.88	16.80 ± 1.81	<0.0001
Time to discharge, hours, mean ± SD	24.12 ± 0.33	29.00 ± 2.94	<0.0001

**Table 2:** Distribution of the type of hernia

Type	Incidence		
Hernia type	Test, n (%)	Control, n (%)	Total, n
Primary ventral	13 (48.14%)	14 (51.8%)	27
Incisional	12 (52.17%)	11 (47.82%)	23
Total	25 (50%)	25 (50%)	50

Postoperative pain was evaluated using the VAS scale at two, six, 12, 24, and 48 hours after surgery (Table 3). Statistically significant differences in VAS scores were observed between the two groups at two, six, and 12 hours, whereas no significant difference was found at the 24- and 48-hour intervals. Rescue analgesic requirements also differed significantly between the groups, with 64% of patients in the Control Group requiring three rescue medications, in contrast to 72% of patients in the Test Group who only needed one rescue medication (p<0.0001).

**Table 3:** Postoperative pain scores VAS VAS: visual analog scale; SD: standard deviation

VAS score	Test Group		Control Group		P-value
	Mean	SD	Mean	SD	
VAS score (1 hour)	4.40	0.50	7.12	0.88	<0.0001
VAS score (2 hours)	4.36	0.49	7.00	1.00	<0.0001
VAS score (3 hours)	3.56	0.58	6.04	1.02	<0.0001
VAS score (6 hours)	3.12	0.53	5.04	0.84	<0.0001
VAS score (12 hours)	2.40	0.50	4.04	0.93	<0.0001
VAS score (24 hours)	2.88	0.73	3.28	0.79	1
VAS score (48 hours)	1.44	0.51	1.72	0.46	1

Ambulation was notably earlier in the Test Group, as patients were able to mobilize at 10.98 ± 0.88 hours compared with 16.80 ± 1.81 hours in the Control Group, which was statistically significant (p<0.0001) (Table 2). Similarly, patients in the Test Group demonstrated a comparatively earlier willingness for discharge at 24.12 ± 0.33 hours, whereas those in the Control Group showed willingness for discharge at 29.92 ± 2.94 hours (p<0.0001) (Table 2).

## Discussion

Reduced postoperative pain is an important component of adequate perioperative care. Effective analgesia is associated with improved surgical outcomes, reduced perioperative stress response, greater patient satisfaction, and decreased opioid consumption with fewer side effects. As minimally invasive techniques become more prevalent, laparoscopic surgery has gained widespread acceptance due to its advantages over open procedures, including less postoperative pain, shorter hospital stays, and quicker recovery times [14-15].

In our study, 50 patients were divided equally into two groups and were assessed for postoperative pain using the VAS score. There were no dropouts, and all patients were followed until discharge. A statistically significant reduction in postoperative pain scores was observed at two, six, and 12 hours in the intervention group. However, this effect was not sustained at 24 and 48 hours, likely due to the pharmacokinetic properties of bupivacaine, which has a half-life of four to eight hours.. These findings align with Fields et al. [15], who demonstrated significant pain reduction at rest within one hour of surgery, though not persisting beyond 24 hours. Importantly, their study also reported a 40% decrease in total morphine requirement among patients receiving a TAP block.

Over the past decade, the TAP block has become a well-established adjunct to multimodal analgesia in abdominal procedures [16-18]. Several studies have documented its ability to lower VAS scores, decrease postoperative opioid and NSAID requirements, and improve patient comfort [19-20]. A meta-analysis by Peterson et al. [21] including seven trials (180 cases, 184 controls) demonstrated an average 24-hour morphine reduction of 22 mg in favor of TAP block recipients. Similarly, Mughal et al. [22], in a randomized trial of laparoscopic inguinal hernia repair, showed significantly reduced rescue analgesic requirements in the TAP group. Further, Susheela et al. [23] reported markedly lower tramadol consumption in patients receiving USG-guided TAP block compared with port-site infiltration following laparoscopic cholecystectomy.

Our study corroborates these findings: 72% of patients in the Test Group required only one rescue analgesic, compared to 64% of controls who required three doses. Effective pain control facilitated earlier ambulation, thereby reducing the risk of complications such as atelectasis, venous thromboembolism, and constipation. Jain et al. [24] reported earlier mobilization with USG-guided TAP block following IPOM repair, with significant differences at both 12 and 24 hours. Sherif et al. [25] also demonstrated significantly earlier ambulation in TAP block patients in bariatric surgery. Likewise, in our cohort, ambulation occurred at 10.98 ± 0.88 hours in the Test Group vs. 16.80 ± 1.81 hours in controls. These outcomes are consistent with Sinha et al. [26], who found earlier readiness for discharge in patients receiving TAP block during LVHR. Our study showed readiness for discharge at 24.12 ± 0.33 hours in the TAP group vs. 29.00 ± 2.94 hours in the control group, a highly significant difference.

The technique itself has evolved from blind to more precise methods. The use of ultrasound and laparoscopy has improved accuracy, safety, and reproducibility. Laparoscopic-guided TAP block, in particular, combines visualization with procedural simplicity, making it effective while requiring less technical expertise [27-29]. Although port-site infiltration remains a recognized and commonly practiced technique [30], the TAP block appears superior in prolonging analgesia and reducing analgesic needs. The physiological impact of uncontrolled perioperative pain in surgical patients is profound and should not be underestimated. Poorly managed pain may increase the risk of complications, delay recovery, and even predispose to chronic pain syndromes. Thus, strategies that provide reliable analgesia with minimal side effects are integral to modern perioperative care. Our findings support the role of laparoscopic-guided TAP block with bupivacaine as a safe, effective, and practical technique, enabling better pain relief, early ambulation, earlier discharge, and ultimately an enhanced quality of recovery.

Limitations

This study, being prospective and observational, has several limitations that should be acknowledged. First, the sample size, while sufficient for preliminary analysis, was relatively limited and confined to a single institution, thereby limiting the external validity and generalizability of the findings. Second, the follow-up period was short, with pain scores assessed only up to 48 hours postoperatively; hence, long-term outcomes such as incidence of chronic pain, late complications, or impact on quality of life could not be evaluated. Third, as is inherent to observational designs, the absence of randomization introduces the potential for selection bias and unmeasured confounders, which may have influenced the observed outcomes. Furthermore, pain assessment was based on the subjective VAS scale, which is inherently variable due to inter-individual differences in pain perception. The study also did not stratify outcomes by important operative factors such as hernia size, number of transfascial sutures, or extent of adhesiolysis, each of which may independently influence postoperative pain. Finally, as the study was conducted at a tertiary academic center by experienced laparoscopic surgeons, the results may not be directly reproducible in peripheral or resource-limited settings.

## Conclusions

In our experience, laparoscopic-guided TAP block emerged as a safe and effective adjunct in the perioperative management of LVHR. Performed under direct laparoscopic visualization, the block allowed for precise placement with minimal risk and had a negligible impact on overall operative time. Its ease of use and minimally invasive nature made it suitable for integration into a multimodal analgesic protocol, resulting in improved pain control, earlier ambulation, and faster readiness for discharge. Equally significant, the block decreased the need for rescue analgesics and enhanced patient satisfaction, contributing to a smoother recovery and supporting the goals of enhanced recovery after surgery (ERAS) protocols. Given its reproducibility, safety profile, and clear clinical benefit in the immediate postoperative period, laparoscopic-guided TAP block can be recommended as a valuable addition to routine practice in ventral hernia repair.
